# A meta-analysis on Omega-3 supplements in preventing recurrence of atrial fibrillation

**DOI:** 10.18632/oncotarget.23783

**Published:** 2017-12-30

**Authors:** Ying Jiang, Hui Ching Tan, Wilson Wai San Tam, Toon Wei Lim, Wenru Wang

**Affiliations:** ^1^ Alice Lee Centre for Nursing Studies, Yong Loo Lin School of Medicine, National University of Singapore, Singapore; ^2^ Department of Nursing, National University Hospital, Singapore; ^3^ National University Hospital, Assistant Professor, Yong Loo Lin School of Medicine, National University of Singapore, Singapore

**Keywords:** Omega-3 polyunsaturated fatty acids, recurrence of atrial fibrillation, meta-analysis

## Abstract

Previous studies had suggested that Omega-3 fatty acids have pleiotropic effects and favourable safety profile, which may potentially increase the efficacy of antiarrhythmic drugs in suppressing atrial arrhythmias through combination therapy. This meta-analysis aimed to determine the effectiveness of using Omega-3 polyunsaturated fatty acids as a sole anti-arrhythmic agent or as an adjunct to existing pharmacological therapies in preventing atrial fibrillation recurrence. Randomized controlled trials published in English, from inception to December 2016, were considered. We searched for published studies in the following electronic databases: Cochrane Central Register of Controlled Trials, PubMed, EMBASE, Medline, Scopus, and Cumulative Index to Nursing and Allied Health Literature. Pooled hazard ratio (HR) and corresponding 95% confidence intervals (CI) for time to first atrial fibrillation recurrence was analysed using a fixed effects model. Four RCTs with 1,268 participants were included in the review. Our results showed that Omega-3 polyunsaturated fatty acid therapy had no effect on preventing atrial fibrillation recurrence compared to control/placebo group (HR: 1.13, 95% CI: 0.96 to 1.33, *p =* 0.14), with no significant heterogeneity found among those studies (*Q* value = 0.15, 9 = 0.99, I^2^ = 0%). Therefore, current evidence does not support treatment benefit of Omega-3 fatty acids in preventing atrial fibrillation recurrence among patients who have not been treated by any conventional reversion treatment, or who have only been treated with pharmacological therapy.

## INTRODUCTION

Atrial fibrillation (AF) is the commonest sustained cardiac arrhythmia affecting approximately 33.5 million individuals worldwide [1]. Although well tolerated in most patients, AF is associated with an increased long-term risk of stroke and all-cause mortality [2]. With an ageing population, the prevalence of AF is expected to increase, therefore, efforts to control this global epidemic is required [3].

Common treatment options to restore sinus rhythm (SR), otherwise known as rhythm control, consist of both pharmacological and non-pharmacological strategies, of which the latter includes electrical cardioversion, catheter ablation and surgical ablation [4]. Although evidence support the efficacy of non-pharmacological methods in rhythm control, the recurrence rate of AF after treatment with non-pharmacological strategies remains high [5]. Compared to pharmacological therapy, the invasive nature of non-pharmacological interventions is associated with poorer tolerability, higher costs [6], and concerns about the risk of life-threatening complications such as pericardial tamponade [4, 7]. As such, antiarrhythmic drugs (AADs) may be preferred in reducing cost and promoting ease of treatment.

AADs can be used alone or as an adjunct therapy to restore and maintain SR [8]. Evidence on the efficacy of AADs as a sole treatment modality is however limited [9], with 1-year success rates ranging between 30% to 65% as compared to 74% to 90% for radiofrequency catheter ablation [7]. This has led to the question of whether it is possible to enhance the efficacy of AADs in long-term suppression of arrhythmias through combination therapy [10].

There has been an increased attention on the potential benefits of non-antiarrhythmic agents such as Omega-3 polyunsaturated fatty acid in AF management. Omega-3 polyunsaturated fatty acid, also commonly known as fish oil products, are concentrated sources of eicosapentaenoic acid and docosahexaenoic acid (DHA). It has been proposed that Omega-3 polyunsaturated fatty acid possess anti-inflammatory properties that can inhibit arrhythmogenic mechanisms [11, 12], as inflammation results in oxidative stress, apoptosis and fibrosis which promote AF substrate formation and arrhythmogeneicity [13].

A number of trials have been carried out to test the potential role of Omega-3 polyunsaturated fatty acid in treating AF, but findings have been inconsistent [14–17]. In the reviews by Mariani *et al.* [18], He *et al.* [19], Cao *et al.* [20] and Liu *et al.* [21], prevention of secondary AF was limited in the treatment modality employed. Electrical cardioversion was the most common treatment and the outcomes were equivocal across all these studies. Given the disadvantages of non-pharmacological approaches, it is thus a suboptimal approach to preventing AF recurrences. Hence, pharmacological options may still be preferred if efficacy can be increased. Apart from the possible antiarrhythmic benefits that Omega-3 polyunsaturated fatty acid can offer to AF patients, it is also worth investigating the effects of Omega-3 polyunsaturated fatty acid supplementation as an adjunct to AAD therapy in preventing AF recurrence. To the best of our knowledge, no review has been done specifically on this topic thus far. Therefore, this review aimed to determine the effectiveness of using Omega-3 polyunsaturated fatty acid as a sole anti-arrhythmic agent or as an added therapy to existing pharmacological therapies in preventing the recurrence of AF. In particular, we examined the rate of AF recurrence, measured by time to first recurrent episode as the primary outcome for this review.

## RESULTS

### Studies identification

**Figure 1:**
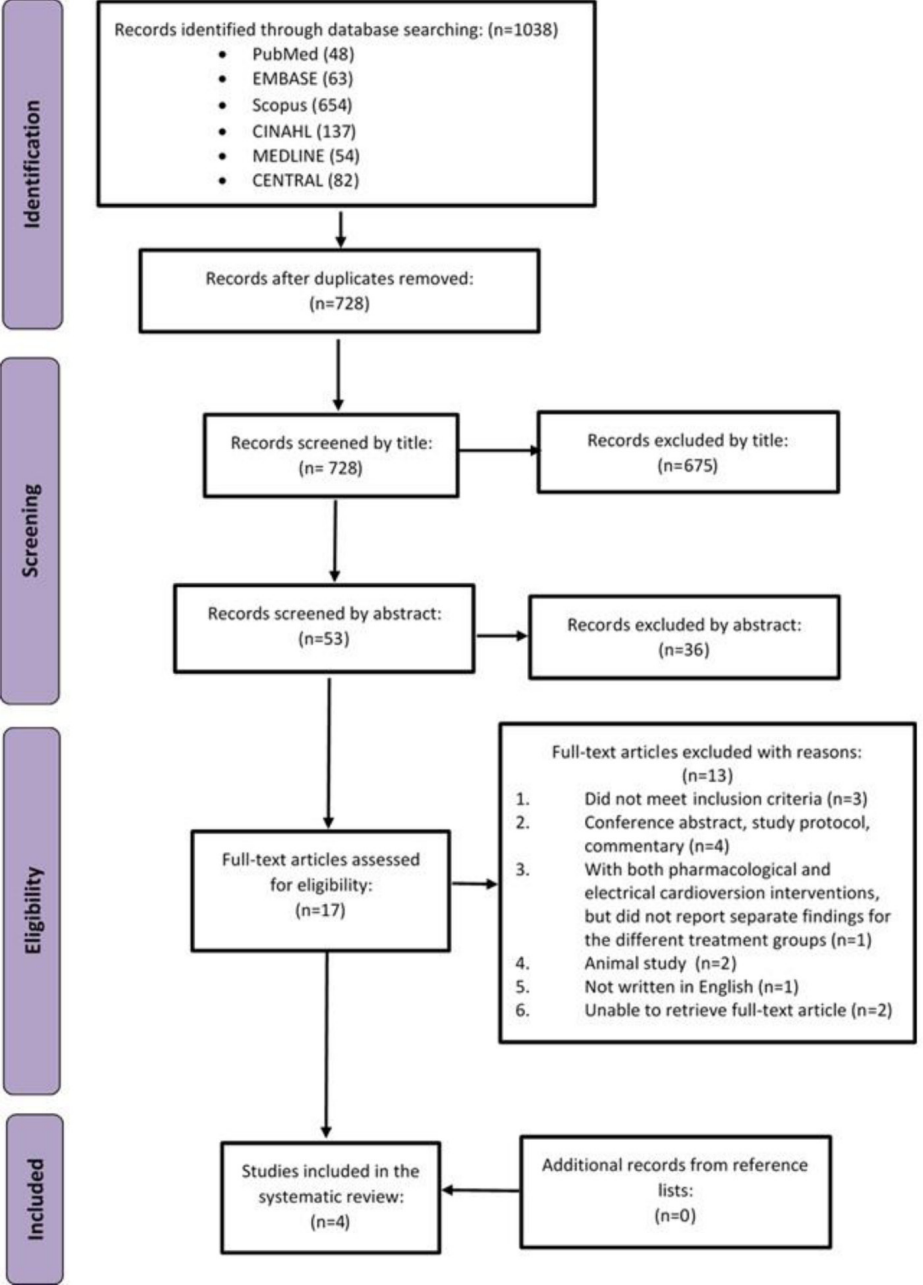
PRISMA flow diagram

The PRISMA flow chart is presented in Figure 1. The initial search across the databases identified 1,038 records. After removal of duplicates, 728 records were screened, of which 675 were excluded after screening the title and 36 were excluded after screening the abstract because they were ineligible. The full texts were retrieved for the remaining 17 records. Subsequently after screening the full texts, 13 articles were excluded for reasons as shown in Figure 1. In total, four RCT studies were included for this meta-analysis [15, 17, 22, 23].

### Description of included studies

The summaries of the included studies are presented in Table 1. Of the four included studies, three were prospective, double–blind, placebo-controlled, parallel arm RCTs [17, 22, 23], and the other study was a prospective, open-label randomized study [15]. The studies were published in the United States, Australia and Canada. In total, there were 1268 participants involved in those studies, of which 56% were male. Participants in the intervention group had a mean age of 64.95 ± 8.76 years old, while participants in the control group had a mean age of 65.30 ± 7.82 years old. Three studies prescribed 4g of omega-3 polyunsaturated fatty acid per day, which contained approximately 1.60 to 1.86 g/day EPA and 0.8 to 1.50 g/day DHA (the active components) as study intervention [17, 22, 23]. The other study by Kumar *et al.* prescribed 6 g/day of omega-3 polyunsaturated fatty acid that contained approximately 1.02 g/day EPA and 0.72 g/day DHA [15]. Two studies had a loading period of 7 days and 21 days [22, 23], while the other two studies did not. The intervention duration and follow up duration varied across the studies, ranging from 6 months or less (stopped when first AF recurrent episode occurred) [17] to up to 16 months [23].

**Table 1:** Summaries of included studies

Authors	Study design	Participants	Intervention	Control	Use of AADs	Intervention description	Control description	Follow-up assessment	Definition of AF	Method to measure	Findings
Kumar *et al.* (2013)	Open-label	AF patients aged ≥60 years with sinoatrial node disease and dual pacemakers	***N*** = 39 **Mean age** = 78 (7)	***N*** = 39 **Mean age** = 77 (8)	Yes	**Omega-3 dosage:** 6 g/day (1.02 g EPA and 0.72 g DHA) **Duration:** 6 month and 12 month **Loading:** No	Not given placebo, but instructions not to commence omega-3 supplements and maintain a stable omega-3 intake	6 month and 12 month	A-A interval irregularity on marker channels and/or fibrillatory activity on EGMs or ECG	Intracardiac EGMs and marker channels. An ECG was performed if a patient was noted to have atrial arrhythmia at the time of interrogation.	At 6 months, time to first episode of AT/AF not significant between 2 groups. At 12 months, rebound increase in AT/AF burden in cross-over patients (*p =* 0.01), reached a level similar to controls (*p =* 0.63), and higher than those who continued with fish oil (*p =* 0.02). Fish oil patients had shorter episodes of AT/AF, with no difference in frequency compared to control.
Darghosian *et al.* (2015)	Double blind	AF patients with a history of at least 2 occurrences of AF or atrial flutter.	***N*** = 126 **Mean age** = 62 (12)	***N*** = 64 **Mean age** = 61 (11)	Yes	**Omega-3 dosage:** 4 g/day (1.86 g EPA and 1.5 g DHA) **Duration:** 6 months or less (AF recurred) **Loading:** No	Corn oil	6 months or until AF recurred	ECG were coded, and evaluated by 2 blinded cardiac electrophysiologists	eCardio Post-Event Recorder (transtelephonic electrocardiographic monitor device), routine transmissions every 2 weeks and if had symptoms suggestive of arrhythmia	No significant difference in time to AF recurrence, even after taking into consideration age, race, gender, randomization stratum (AADs), CHD, CHF, and duration of AF (HR = 1.20, 95% CI: 0.76-1.90, *p =* 0.438). Intervention was not associated with clinically important effects on concentrations of markers of inflammation and oxidative stress either.
Kowey *et al.* (2010)	Double blind	Patients with symptomatic paroxysmal or persistent AF	***N*** = 266^a^ **Mean age** = 60.0 (13.56)	***N*** = 276^a^ **Mean age** = 61.9 (11.57)	No	**Omega-3 dosage**: 4 g/day (1.86 g EPA and 1.38 g DHA) **Duration:** 6 months **Loading:** 8 g/day for the first 7 days	1g corn oil	6 months	no mention	Biweekly transtelephonic monitoring	Among patients with paroxysmal AF, 6 months treatment with prescription omega-3 did not reduced recurrent AF compared with placebo group
Nigam *et al.* (2014)	Double blind	Patients with symptomatic paroxysmal or persistent AF	***N*** = 153 **Mean age** = 60 (12)	***N*** = 163 **Mean age** = 62 (13)	No	**Omega-3 dosage:** 4 g/day (1.6 g EPA and 0.8 g DHA) **Duration:** 6 months to 16 months (or till first AF) **Loading:** 4 g/day for the first 3 week	1 g safflower oil	6 months to 16 months (or less, till first AF)	Asymptomatic or symptomatic AF recurrence lasting ≥ 30 s	Monitor weekly transtelephonic monitor transmissions, 12 lead ECG or implanted device.	Omega-3 did not reduce AF recurrence in patients with a history of AF not receiving conventional AA therapy.

^a^paroxysmal AF stratum (who had never treated by long-term pharmacological or electrical therapy)

### Risk of bias

**Figure 2:**
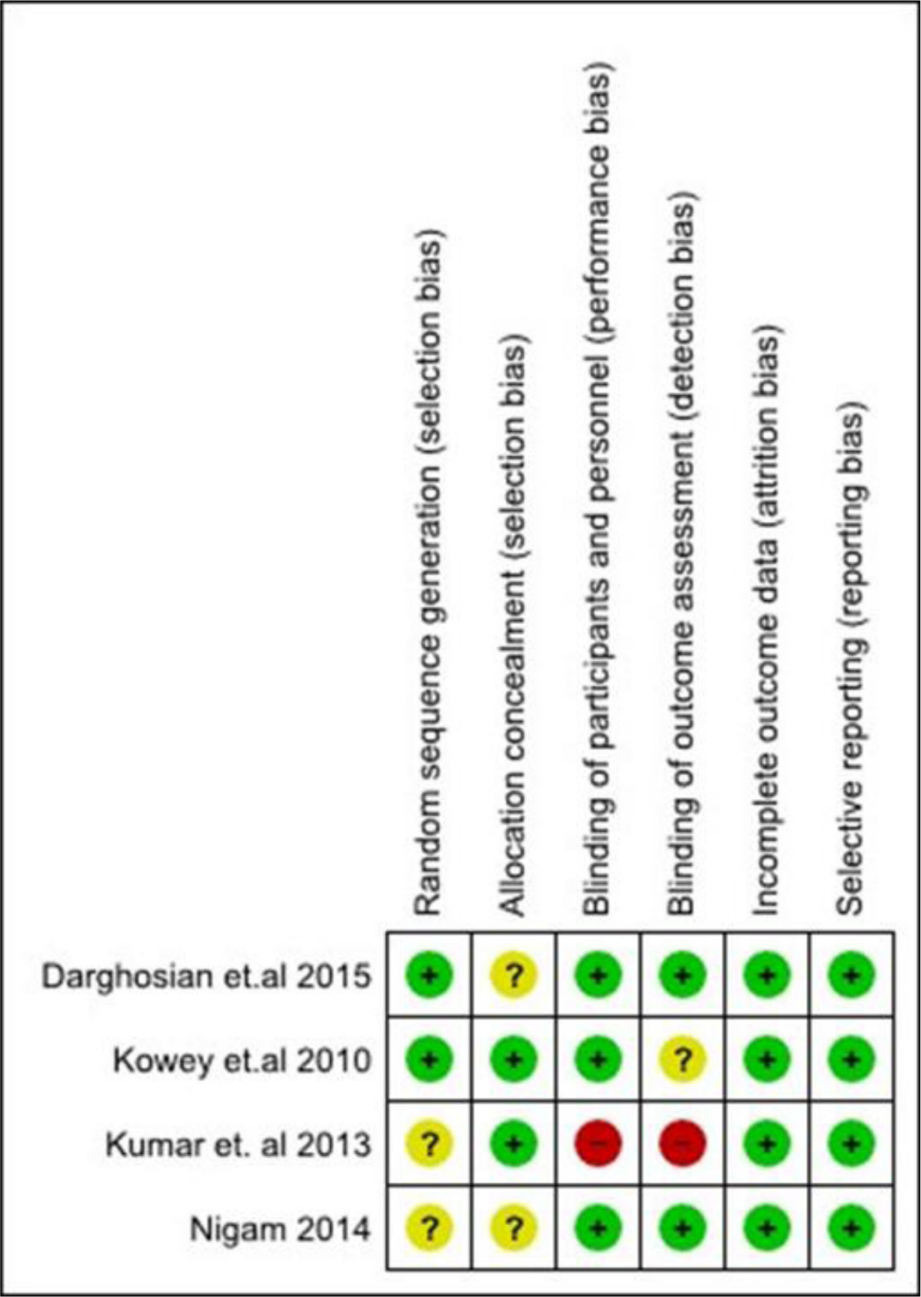
Risk of bias summary

Figure 2 is a summary of the risk of bias assessment for the included studies. Overall, the risk of bias among these studies was low. However, half of the studies did not provide details on the methods for random sequence generation or mention the implementation of allocation concealment. One study adopted an open label design and outcome data were retrieved in an un-blinded fashion [15], therefore incurring a higher risk of bias than the other three studies. All the studies reported the use of intention to treat to manage missing values and reported their outcomes of interest completely.

### Meta-analysis

**Figure 3:**
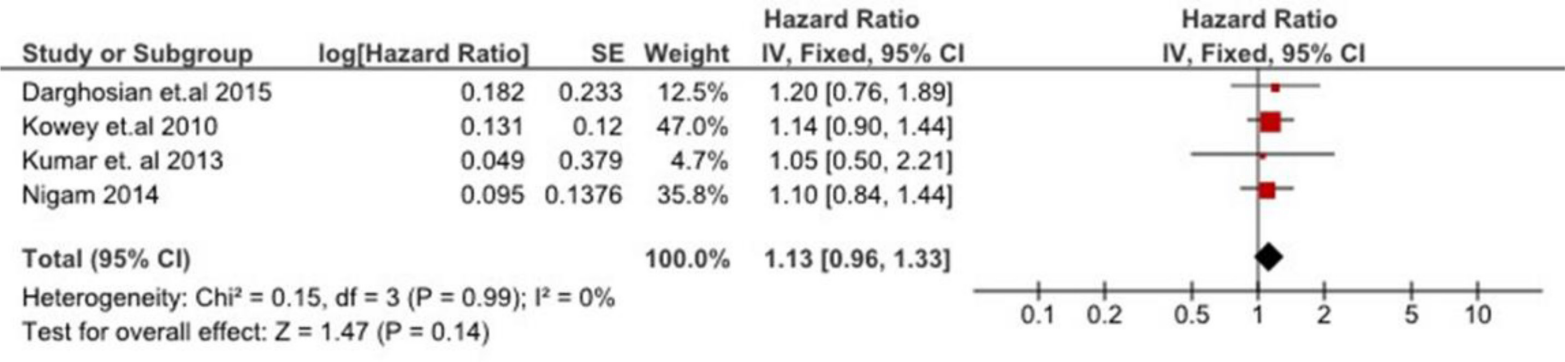
Effect of omega-3 polyunsaturated fatty acid on time to first AF recurrent episode

Figure 3 shows a forest plot of the pooled hazard ratio (HR) of AF recurrence in the participants who received omega-3 polyunsaturated fatty acid compared to those who did not. When results were combined, omega-3 polyunsaturated fatty acid therapy had no effect in preventing AF recurrence compared to control/placebo group (HR: 1.13, 95% CI: 0.96 to 1.33, *p =* 0.14), with no significant heterogeneity found among those studies (*Q* value = 0.15, 9 = 0.99, I^2^ = 0%).

In fact, none of the included RCTs showed a significant difference in time to first AF recurrence between the intervention and control group. Among the low-risk patients with paroxysmal or persistent AF who have never received conventional anti-arrhythmic therapy, Omega-3 fatty acids did not show any rhythm-control benefits [23]. In the high-risk pacemaker population, long-term high dose Omega-3 fatty acids use was associated with shorter episodes of AF recurrence compared to the control group, but no differences in the frequency of recurrent episodes was found [15]. Furthermore, two studies reported that Omega-3 fatty acids did not result in any significant changes in the concentrations of inflammatory or oxidative stress markers as compared to controls [17, 23]. Moreover, the high dose of Omega-3 fatty acids was poorly tolerated by the treatment group [15].

## DISCUSSION

To the best of our knowledge, this is the first meta-analysis to examine the effects of Omega-3 fatty acids as a sole anti-arrhythmic agent or as a supplementation to AADs in preventing AF recurrence. The review included four RCTs involving 1,268 participants across three countries. Result revealed that Omega-3 polyunsaturated fatty acid has no beneficial effect on AF recurrence among patients who have not been treated by any conventional rhythm control treatment, or who have only been on pharmacological therapy.

An earlier cohort study of 4815 participants had found that consumption of tuna and other broiled or baked fish with increased plasma phospholipid long-chain n-3 fatty acids level was linked to a lower incidence of AF [24]. However, subsequent cohort studies with larger sample sizes failed to replicate the result [25, 26]. Similarly, although animal experimental studies suggest that omega-3 fatty acids possess anti-arrhythmic properties of [27–30], results from clinical trials have been disappointing.

Our results are in line with previous systematic reviews and meta-analyses examining the effects of Omega-3 polyunsaturated fatty acid on the secondary prevention of AF after electrical cardioversion [18–21] and in AF prevention in patients undergoing heart surgery [18, 31]. The discrepancies between experimental studies and clinical trials could be partly attributed to publication bias, in which positive results of animal studies were more likely to be published compared to those with negative findings [20]. Other plausible reasons may include a chance effect of small sample size from these earlier animal studies (sample sizes ranging from 8 to 24) [27, 28, 30], the significantly much smaller remodeling capacity of an animal heart [19], the higher doses used in the animal experiments and the different route of administration (intravenous vs oral) resulting in discrepant bioavailability [27].

Several mechanisms have been recognized in the pathogenesis of AF, and among these, the role of inflammation and oxidative stress is believed to be fundamental in the pathophysiology of atrial remodeling [17, 32, 33]. Evidence from previous research suggested that Omega-3 fatty acid lowers systematic inflammation and oxidative stress by preventing the catalysis of arachidonic acid into potent inflammatory aggregators and suppressing important inflammatory mediators, such as interleukin (IL)-1β, tumor necrosis factor and IL-6 [11, 12]. However, these findings have not been consistent. Despite the relatively large sample sizes and high doses of Omega-3 fatty acid, Darghosian *et al.* [17] and Nigam *et al.* [23] found that Omega-3 fatty acid had no effect in reducing inflammation or oxidative stress as measured though serum biomarkers [17], c-reactive protein (CRP) and myeloperoxidase (MPO) [23] compared to controls. Therefore, the inability of Omega-3 fatty acid to reduce AF recurrence could be due to the absence of a clinically relevant effect on the underlying pathophysiological processes [23].

In addition, although it has been found that omega-3 fatty acids may have better antiarrhythmic potential when it is incorporated into cell membranes [34], the complex interaction between circulating and tissue levels of omega-3 fatty acids and its resulting effects are not fully understood [21]. Metcalf *et al.* [35] suggested that omega-3 supplementation in human subjects gave a slow and gradual increase in tissue levels over 2 months, while plasma levels rose shortly and remained constant. Therefore, a short course of supplementation may not have allowed sufficient incorporation of omega-3 fatty acids into cardiac tissues to show an effect [21]. In the study by Kowey *et al.* [22] a loading phase of 7 days was included, but it was noted that nearly half of the participants who received omega-3 fatty acids experienced AF recurrence within the 56 days (first 2 months) of omega-3 consumption, which may suggest insufficient time for building up tissue levels.

## LIMITATIONS

Our review has several limitations. Firstly, we limited our review to only one outcome, which was time to first AF recurrence as measured by hazard ratio. Therefore, potential benefits of Omega-3 fatty acid in AF prevention measured by other outcomes or clinical indicators, such as the frequency of AF episodes and the duration of each episode, were not captured. Secondly, we only included randomized studies in the review and articles written in English. As a result, only four studies were included, and the population in these studies were strictly defined. This may not have been a true reflection of the clinical profile of all AF patients in the community. Therefore, caution should be exercised when extrapolating our results to wider clinical settings. Thirdly, because of the limited number of studies, when analyzing the effect of Omega 3 fatty acids in combination with ADDs, we could not stratify the effect based on the types of ADDs.

## CONCLUSIONS AND CLINICAL IMPLICATION

Omega-3 fatty acid, with its pleiotropic effects and favorable safety profile has been proposed as a potential adjunct to augment the efficacy of AADs for suppressing arrhythmias. However, this present meta-analysis showed that Omega-3 fatty acid consumption either as a supplement to AADs or as a sole anti-arrhythmic agent is not associated with a reduction in AF recurrence. Therefore, there is insufficient evidence to support its routine use in either role.

## METHODS

The study followed the preferred reporting items for systematic reviews and meta-analyses (PRISMA) guideline [36].

### Inclusion criteria

We included studies that were randomized controlled trials (RCTs), which met the following inclusion criteria: 1) Study participants were adult patients with a confirmed diagnosis of AF. 2) The arrhythmia had been treated by either exclusively pharmacological means or with no treatment prior to the point of study enrolment, and patients were in sinus rhythm before study initiation. 3) Studies that evaluated the effects of Omega-3 fatty acids as a sole anti-arrhythmic agent or as a supplement to AADs in preventing AF recurrence. 4) Studies that separated participants into at least one group receiving Omega-3 fatty acids treatment and one group receiving a placebo treatment or no treatment. 5) Studies that included the recurrence of AF as the study outcome.

### Exclusion criteria

We excluded studies in which the intervention was a form of primary prevention in patients who had not been diagnosed with AF and studies with overlapping study populations (such as participants had been previously treated with drugs or electrical cardioversion therapy), but did not report separate findings for the different treatment groups.

### Search strategy

The search strategy comprised of a three-step approach. First, a preliminary search in Cochrane and MEDLINE was conducted to identify permutations of text words and index terms contained in the title and abstracts used to describe articles. Second, a literature search was conducted by the first two authors (JY and HC) using the identified keywords and index terms on the following databases to identify the published studies: Cochrane Central Register of Controlled Trials (CENTRAL), PubMed, EMBASE, Medline, Scopus, and Cumulative Index to Nursing and Allied Health Literature (CINAHL). The keywords used included: omega 3, polyunsaturated fatty acid*, PUFA, n-3 fatty acid*, eicosapentaenoic acid*, EFA, alpha-linolenic acid*, docosahexaenoic acid*, DHA, fish oil, atrial fibrillation, atrial arrhythmia*, auricular fibrillation*, auricular arrhythmia*, atrium fibrillation*, atrium arrhythmia*. The search strategy is presented in the Appendix. The search was limited to English studies conducted from inception to December 2016. Lastly, the reference lists from the included articles were hand searched to identify other potentially relevant articles.

### Study selection

Two independent authors (JY and HC) screened the title and abstract of the records retrieved from the databases to identify the potentially relevant studies. After which, the full texts of potentially relevant studies were retrieved and critically appraised based on the inclusion and exclusion criteria before being included in the review. Disagreements were resolved through discussion between the two primary reviewers.

### Primary outcomes

The recurrence of AF, measured as time to first recurrent episode was the primary outcome for this review.

### Data extraction

Study characteristics, such as research design, participants’ characteristics, intervention description, reported outcomes, and statistical parameters, such as sample size, hazard ratio were extracted using a structured data extraction form adapted from the Cochrane Handbook for Systematic Reviews of Interventions [37]. Two reviewers (JY and WW) reviewed the studies independently, and summary tables were discussed to ensure the accuracy and relevance of the extracted data.

### Assessment of risk of bias

Two independent assessors (JY and WW) assessed the risk of bias of the included studies based on the Cochrane Collaboration tool for assessment of risk of bias described in the *Cochrane Handbook for Systematic Reviews of Interventions* [37]. The following criteria were assessed: (1) random sequences generation (selection bias), (2) allocation concealment (selection bias), (3) blinding of participants and personnel (performance bias), (4) blinding of outcome assessment (detection bias), (5) incomplete outcome data (attrition bias), and (6) selective reporting (reporting bias). Differences between the two reviewers were resolved through discussion to reach a consensus.

### Data syntheses and analyses

AF recurrence as measured by time to first recurrent episode was expressed in Hazard Ratio (HR) with 95% CI. The natural logarithm and the standard error of the HR were computed for each study. The fixed effect and random-effects models using inverse variance approach were adopted in the absence and presence of heterogeneity respectively. Heterogeneity among the studies was evaluated by the Cochran’s *Q* test and I^2^ statistic. The *p*-value of the Cochran’s *Q* test was set at <0.1 to suggest statistical heterogeneity. I^2^ statistic was used to estimate the percentage of variation across the studies that is due to heterogeneity, with 75%, 50%, 25%, or 0% indicating high, moderate, low, or no heterogeneity, respectively [38]. The overall effect was assessed by Z-statistic. The significance level was set at p < 0.05. Computations for the meta-analysis were conducted using the RevMan software (Review Manager Version 5.3 for Windows from The Nordic Cochrane Centre, The Cochrane Collaboration, 2014) [37]. Subgroup analysis would be conducted to explore the source of heterogeneity when necessary.

## SUPPLEMENTARY MATERIALS



## Abbreviations

AADs: antiarrhythmic drugs
AF: atrial fibrillation
CENTRAL: Cochrane Central Register of Controlled Trials
CINAHL: Cumulative Index to Nursing and Allied Health Literature
DHA: docosahexaenoic acid
HF: hazard ratios
RCTs: Randomized controlled trials
