# Supplementary material for: A meta-analysis on Omega-3 supplements in preventing recurrence of atrial fibrillation
Source: Oncotarget. 2017 Dec 30;9(5):6586–94. doi: 10.18632/oncotarget.23783 (PMC5814234; doi:10.18632/oncotarget.23783)
Supplement: Supplementary file 1 [file oncotarget-09-6586-s001.pdf]

A meta-analysis on Omega-3 supplements in preventing recurrence of atrial fibrillation

SUPPLEMENTARY MATERIALS

Appendix: Search strategy

Search strategy

PubMed (Medline)

#1 "Atrial Fibrillation"[Mesh]
#2 "Atrial fibrillation" OR "atrial fibrillation*" OR "atrial arrhythmia*" OR "auricular fibrillation*" OR "auricular arrhythmia*" OR "atrium fibrillation*" OR "atrium arrhythmia*"
#3 #1 OR #2
#4 "Fatty Acids, Omega-3"[Mesh]
#5 "omega-3" or "omega 3" or "polyunsaturated fatty acid*" or "n-3 polyunsaturated fatty acid*" or "n 3 polyunsaturated fatty acid*" or "PUFA" or "n-3 PUFA" or "n 3 PUFA" or "n-3 fatty acid*" or "n 3 fatty acid*" or "long chain fatty acid*" or "unsaturated fatty acid*" or "essential fatty acid*" or "eicosapentaenoic acid*" or "EFA" or "alpha-linolenic acid*" or "ALA" or "α-linolenic acid*" or "α linolenic acid*" or "docosahexaenoic acid*" or "DHA" or "linseed oil" or "flaxseed oil" or "fish oil"
#6 #4 OR #5
#7 [#3 AND #6] [Title/Abstract]
